# Can Communicating Personalised Disease Risk Promote Healthy Behaviour Change? A Systematic Review of Systematic Reviews

**DOI:** 10.1007/s12160-017-9895-z

**Published:** 2017-03-13

**Authors:** David P French, Elaine Cameron, Jack S Benton, Christi Deaton, Michelle Harvie

**Affiliations:** 1 Manchester Centre of Health Psychology, School of Health Sciences, University of Manchester, Coupland 1 Building, Manchester, M13 9PL England; 2 School of Clinical Medicine, Forvie Site, University of Cambridge, Cambridge, CB2 0SR England; 3 Nightingale Centre, University Hospital South Manchester, Manchester, M23 9LT England

**Keywords:** Systematic review, Risk communication, Behaviour change, Behaviour

## Abstract

**Background:**

The assessment and communication of disease risk that is personalised to the individual is widespread in healthcare contexts. Despite several systematic reviews of RCTs, it is unclear under what circumstances that personalised risk estimates promotes change in four key health-related behaviours: smoking, physical activity, diet and alcohol consumption.

**Purpose:**

The present research aims to systematically identify, evaluate and synthesise the findings of existing systematic reviews.

**Methods:**

This systematic review of systematic reviews followed published guidance. A search of four databases and two-stage screening procedure with good reliability identified nine eligible systematic reviews.

**Results:**

The nine reviews each included between three and 15 primary studies, containing 36 unique studies. Methods of personalising risk feedback included imaging/visual feedback, genetic testing, and numerical estimation from risk algorithms. The reviews were generally high quality. For a broad range of methods of estimating and communicating risk, the reviews found no evidence that risk information had strong or consistent effects on health-related behaviours. The most promising effects came from interventions using visual or imaging techniques and with smoking cessation and dietary behaviour as outcomes, but with inconsistent results. Few interventions explicitly used theory, few targeted self-efficacy or response efficacy, and a limited range of Behaviour Change Techniques were used.

**Conclusions:**

Presenting risk information on its own, even when highly personalised, does not produce strong effects on health-related behaviours or changes which are sustained. Future research in this area should build on the existing knowledge base about increasing the effects of risk communication on behaviour.

**Electronic supplementary material:**

The online version of this article (doi:10.1007/s12160-017-9895-z) contains supplementary material, which is available to authorized users.

The global burden of disease is increasingly due to non-communicable diseases such as cardiovascular disease, diabetes, and cancer at least partly caused by health-related behaviours e.g. smoking and lack of physical activity [1]. Two commonly used approaches to preventing such diseases both involve the estimation of personalised disease risk. First, personalised risk scores are generated to triage prevention therapies to groups with optimal risk benefit ratios, e.g. statins for individuals identified with 10 year risk of cardio-vascular disease greater than 10% [2] and tamoxifen or raloxifene for women with 10 year risk of breast cancer greater than 5% [3]. Second, personalised risk scores are communicated to patients with the expectation that telling people that they are at high risk will motivate them to engage in health-related behaviour changes to reduce their risk [4].

For both of these reasons, the assessment and communication of personalised disease risk is currently happening on a large scale. Examples include risk of cardio-vascular disease based largely on physiological markers and family history, e.g. QRisk [5], breast cancer risk estimates derived mainly from family history assessments, hormonal risk factors and weight [6], and coronary artery calcification using non-invasive visual imaging techniques [7]. The assessment and communication of personalised risk estimates is likely to increase with the proliferation of inexpensive DNA-based tests for common genetic variants that contribute to many non-communicable multifactorial diseases. Accordingly, it is important to evaluate the extent to which these risk estimation and communication programmes produce changes in health-related behaviours, and to determine which approaches to communicating risk are most likely to bring about this behaviour change.

The current state of knowledge is good in terms of communicating risk information that is not personalised to the individual, e.g. that smoking is generally harmful [8–10]. A recent systematic review of risk communication studies found that where interventions produced a significant increase in risk appraisal relative to control participants, there was a mean increase of d=0.23 on subsequent behaviour, across 93 studies [9]. Further, in line with theory [11], effect sizes of subsequent behaviour were much larger (d=0.45) when response efficacy and self-efficacy were also increased. Response efficacy refers to a person’s belief that changing their behaviour (e.g. increasing physical activity) will reduce risk, and self-efficacy refers to a person’s belief that they are capable of changing the relevant behaviour. There is also good evidence on the most effective means of increasing self-efficacy, at least for some behaviours such as physical activity [12].

Despite this good state of knowledge regarding the most effective ways of communicating information about non-personalised risk to change behaviour, it is unclear to what extent self-efficacy and response efficacy have been targeted in interventions involving personalising risk information. Further, it is not clear which behaviour change techniques to address risk appraisals and efficacy appraisals have been used in such interventions [13]. More generally it is not clear about the extent to which explicit theory has been used, e.g. to select intervention contents, constructs that interventions target, or measures of these constructs, or to inform further theorising about how best to change these health-related behaviours in light of study findings.

Research in the area of personalised risk communication is less developed than research on communicating information on general disease risk information that is not personalised to the individual, but growing rapidly. Consequently, it is difficult for decision makers to maintain familiarity with all relevant findings regarding how best to communicate personalised risk information to bring about behaviour change. This is increasingly a typical state of affairs in science, which has resulted in an increase in the conduct of systematic reviews [14].

Previous systematic reviews of personalised risk communication have overlapped in terms of the condition for which risk is being communicated, the behaviour to reduce this risk, and the nature and source of the personalised risk information. Given the growth in systematic reviews, more recently systematic reviews of systematic reviews have been increasingly employed to compare and synthesise reviews and provide up-to-date summaries of the state of knowledge within specific areas [15]. Such reviews can generate broad overviews not provided by more focussed systematic reviews, highlighting differences as well as commonalities to identify particularly promising approaches. Following this reasoning, the present systematic review of systematic reviews summarised and appraised recent reviews of the effects of communicating personalised disease risk information on individual health-related behaviours.

Our overall aim was to provide an overview of current knowledge from systematic reviews of randomised and non-randomised trials, on the extent to which communicating personalised risk information to adult individuals results in changes in four key health-related behaviours (smoking, alcohol consumption, physical activity and diet) compared to no personalised risk information. One secondary aim was to assess the extent to which this varies by (a) the nature and source of risk information provided, e.g. genetic tests, imaging, etc., (b) the nature of the behaviour that may change, e.g. smoking, physical activity, etc., and (c) the condition for which behaviour may affect risk, e.g. various cancers, diabetes, etc. A further secondary aim was to describe the primary literature on personalised risk communication in relation to factors that have been found to be important for studies of non-personalised disease risk communication, namely (a) use of self-efficacy and response efficacy in interventions and measures, (b) behaviour change techniques used in the interventions, and (c) the explicit use of theory to inform and develop interventions.

## Methods

### Inclusion/Exclusion Criteria

Systematic reviews had to report on primary studies involving adult participants, who did not already have the condition for which their personalised risk was estimated and were not selected on the basis of another clinical condition, to ensure that the results related to primary prevention.

Included primary studies had to report on the effects of communicating personalised disease risk information about common multifactorial conditions that are reliably linked to health-related behaviour, e.g. cardio-vascular disease, diabetes, cancer, and dementia. Personalised disease risk had to be either (a) an estimate of personal susceptibility typically communicated as a score, percentage or category such as ‘low’ ‘medium’ or ‘high’, or (b) feedback on current physiological status that indicates the precursor to clinical disease, e.g. images showing atherosclerosis. Note that participants in intervention studies may have received other interventions, e.g. education or counselling as well as risk estimates. We did not include risk estimates that were based purely on current health-related behaviours, e.g. smoking status, or analogue studies where participants were asked to consider their reactions to hypothetical risk, nor studies where participants received information about actual disease status rather than risk.

People who were allocated to comparison groups in primary studies must not have received personalised risk information (e.g. usual care, no intervention, general disease risk information, or other non-personalised intervention). Eligible study designs were randomised controlled trials (RCTs) and non-randomised controlled trials (NRCTs).

Only studies examining effects on smoking, alcohol consumption, physical activity and diet were included. We did not include studies reporting only the health or physiological outcomes associated with these behaviours, such as change in body weight or blood pressure. Studies of the impact of risk communication on other health behaviours, i.e. (a) risky sexual behaviour, (b) use of tanning booths or sunscreen use, or (c) uptake of screening were not included, as they related to (a) communicable diseases, (b) diseases with one predominant cause rather than being multifactorial, and (c) detection rather than prevention of disease.

The reviews themselves had to employ systematic methods, involving a minimum of conducting online electronic literature searches, and applying suitable inclusion and exclusion criteria for primary studies. We included only systematic reviews that had the explicit aim of investigating the effects of risk communication, to avoid the necessity of searching through reviews that included all interventions, not just those concerned with risk communication. Further, the systematic reviews had to provide sufficient results of primary studies that were RCTs or NCRTs rather than report results solely in combination with studies that were not eligible such as before and after studies. The results of the systematic review must have been presented in a quantitative format, e.g. as effect sizes or significance levels.

Only systematic reviews published in English were included. We included systematic reviews published from 2008 onwards, to avoid inclusion of redundant older reviews alongside newer reviews in the same area. For example, [16] covers highly similar ground to the later review [17].

### Search Strategy

Medline, Cochrane Database of Systematic Reviews, CINAHL Plus and PsycInfo were searched in January 2016. Search terms were adapted from previous reviews [18, 19], and were initially developed for use in Medline (presented in Appendix One). The search strategy was then modified for use with the other three databases. We searched for additional reviews using backward and forward citation searching from included reviews and other relevant articles.

The titles and abstracts of all papers initially retrieved were screened by two authors (EC and MH), with 99.7% (2735/2742) agreement. Those selected were subjected to full text assessment by three authors (DF, MH and CD), with mean 75% agreement, and consensus on inclusion reached through discussion.

### Quality Assessment

The quality of included systematic reviews were scored by two authors using the Amstar scoring system [20]. Agreement on Amstar scores (± 1 point) was 56%, and differences in scores were resolved through discussion.

### Coding of Primary Studies

Primary studies were coded, in relation to six characteristics: (a) what was the nature and source of the risk information communicated, e.g. spirometry, genetic testing, etc.; (b) which of the four behaviours were examined; (c) to which medical condition the risk information communicated was related; (d) the extent to which self-efficacy and response efficacy were described as being targeted and measured post-intervention; (e) use of behaviour change techniques, assessed using a standardised taxonomy [13]; (f) the theoretical grounding of intervention and study, coded using a shortened version of a standardised coding scheme [21].

### Evidence Synthesis

In line with recommendations for systematic reviews of systematic reviews [15], our analysis was mainly descriptive. We described (a) the scope of the included systematic reviews, (b) the quality of the reviews, (c) overlap between studies included in multiple reviews, and then (d) summarised the findings and conclusions of each systematic review. The commentary summarising the findings of the systematic reviews was mainly concerned with the evidence regarding whether communicating personalised risk information result in changes in health-related behaviours. These findings were discussed: (i) overall evidence of effectiveness (ii) in relation to different sources of risk information, (iii) in relation to different health-related behaviours, and (iv) in relation to different medical conditions.

A summary is then presented of characteristics of the individual studies contained within the systematic reviews identified, with a particular focus on (a) whether self-efficacy and response efficacy were targeted by the interventions; (b) which behaviour change techniques were included in interventions; and (c) use of theory in these primary studies. Given that some primary studies were reported in multiple reviews (quantified below), it was not appropriate to quantitatively combine findings across the reviews [15]. It should also be noted that the reporting of results in several primary studies included in these systematic reviews was not done sufficiently well to allow aggregation across primary studies.

## Results

**Fig. 1:**
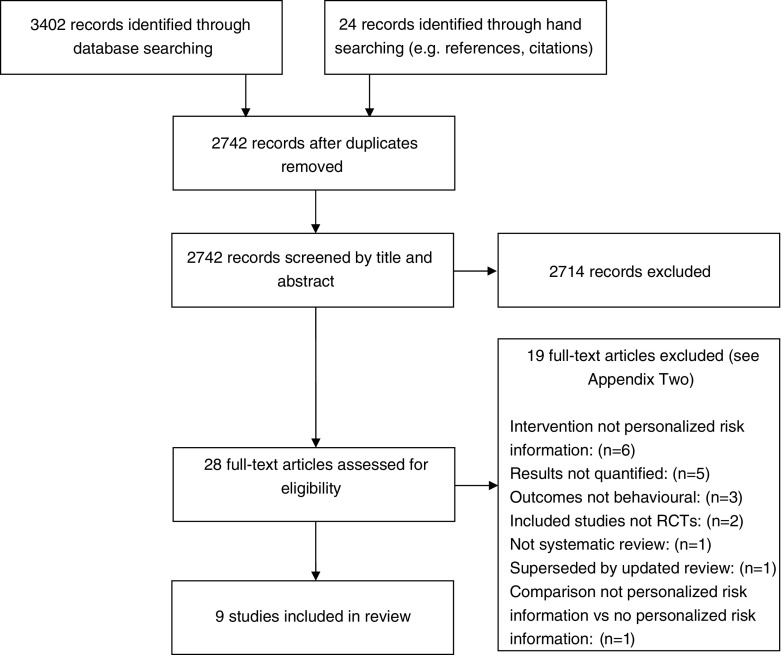
PRISMA flowchart of paper selection process

An electronic literature search conducted in January 2016 identified 2718 unique abstracts, and 24 more were identified through backward and forward citation searching. Following a two-stage screening process, nine systematic reviews were deemed eligible for inclusion (see Table 1 and Fig. 1). The 19 papers excluded at the full-text screening stage are reported in Electronic Supplementary Material 1.

**Table 1:** Summary table indicating the scope of included reviews in the current systematic review of reviews

Author (date)	Intervention	Outcome and participants	Search strategy	Number of studies included	Total N
Bize (2012) [17]	Biomedical risk assessment (various methods)	Smoking cessation in primary prevention patients	4 databases (August 2012): Cochrane, MEDLINE, EMBASE, PsycINFO. Search strategy provided	15 trials [16 interventions]: 4 carbon monoxide testing only; 4 carbon monoxide testing + spirometry; 3 spirometry only; 2 carotid ultrasound; 3 genetic susceptibility	6673
DeViron (2012) [25]	Genetic testing for smoking related disease risk	Smoking cessation in general population	6 databases (August 2011): PubMed, EMBASE, Scopus, Web of Science, PsycINFO, Toxnet). No language restriction, forward citation searching, published studies only, search strategy provided	5 trials (3 RCTs and 2 non-RCT, involving sequential allocation): 4 genetic testing only; 1 genetic testing + carbon monoxide testing	2304
Hackam (2012) [28]	Non-invasive cardiovascular imaging (various methods)	Behaviours (smoking [*n*=4], diet [*n*=1], physical activity [*n*=1])	11 databases searched (November 2010): including Cochrane, MEDLINE, EMBASE, Web of Science. Language restrictions unclear, forward citation searching, grey literature included, search strategy not provided	4 trials: 3 coronary calcification, 1 carotid atherosclerosis	709
Hollands (2010) [27]	Feedback of medical imaging results (various methods)	Health-related behaviours (smoking [*n*=3], diet [=1], physical activity [*n*=1]) to reduce risk (NB several studies excluded from present analyses as behaviours not relevant)	7 sources searched (September 2009): Cochrane, MEDLINE, EMBASE, CINAHL, PsycINFO, metaRegister of RCTs, ProQuest. Language restrictions unclear, forward citation searching, grey literature included, search strategy provided	4 trials (2 ultrasound, 2 computed tomography feedback)	684
Marteau (2010) [19]	Communicating DNA-based risk estimates	Health-related behaviours (smoking [*n*=5], diet [=2], physical activity [*n*=2])	5 sources searched (April 2010): Cochrane, MEDLINE, EMBASE, CINAHL, PsycINFO. No language restrictions, forward citation searching, grey literature included, search strategy provided	7 trials: 6 genetic testing only; 1 genetic testing + carbon monoxide testing	2762
Author (date)	Intervention	Outcome and participants	Search strategy	Number of studies included	Total N
Rodondi (2011) [26]	Feedback of noninvasive atherosclerosis screening	Health-related behaviours (smoking [*n*=7], diet [=6], physical activity [*n*=5]) to reduce risk in people without pre-existing CVD	2 sources searched (September 2009): Cochrane and MEDLINE. All languages, backward citation searching, no grey literature included, no search terms provided	9 trials (3 RCTs, 6 non-RCTs) – 6 computed tomography, 2 carotid ultrasound, 1 total body scan	3340 (*n*=659 from RCTs, *n*= 2681 from non-RCTs)
Sheridan (2010) [22]	Providing CHD risk estimation (including counselling/ education)	Health-related behaviours (smoking [*n*=6], diet [=3], physical activity [*n*=5]) for primary prevention in people without CHD	4 databases searched (Cochrane, PsycINFO, CINAHL, Medline) in Dec 2008. English language only, backward citation searching, no grey literature, no search terms provided	7 trials– based on risk calculators [6] including Framingham [3] based scores, and one where no formal calculator was used.	18,057
Smerecnik (2012) [24]	providing genetic testing for cancer risk	Smoking cessation in general population	8 databases searched (PubMed, EMBASE, ERIC, PsycINFO, PsychArticles, CiNAHL, socINDEX, Google Scholar) – unclear when. English language only, backward citation searching, no grey literature, search terms partly provided	5 RCTs (same studies as in DeViron, 2012)	2274
Usher-Smith (2015) [23]	Effects of providing cardiovascular risk estimates only	Health-related behaviours (smoking [*n*=3], diet [*n*=1], physical activity [*n*=1], alcohol consumption [*n*=2]) for primary prevention in people without CHD	2 databases searched (Medline and PubMed) in Jun 2013. No language restrictions, backward citation searching, published studies only, search strategy provided	3 RCTs, each using different CVD risk calculator	2784

### Quality of Included Reviews

The nine included systematic reviews were generally good quality according to the AMSTAR system [20]. Scores for the eleven AMSTAR criteria are presented in Electronic Supplementary Material 2. Total scores for the nine reviews ranged from seven [22] to 11 [19]. The most common limitations of the reviews were not providing lists of included and excluded studies (six reviews failed to do this) and publication bias not being assessed (six reviews failed to do this).

### Overlap between Reviews

The reviews varied in number of studies included, from only three primary studies [23] to 15 primary studies [15], with a median of five studies included. However, there was considerable overlap in the studies included in the reviews, with only 36 unique studies being covered by the nine reviews. The majority of the primary studies were included in one review only (*n*=25), but the remainder were included in two reviews (*n*=3), three reviews (*n*=4) or four reviews (*n*=4).

### Does Communicating Personalised Risk Information Result in Changes in Health-Related Behaviours?

**(a) Overall**. The main results of each review were summarised in Table 2, along with the review main conclusions and a summary of assessments of risk of bias, and heterogeneity and sensitivity analyses. The clear overall picture that emerges from consideration of review conclusions is that none of the nine reviews concluded that effects on behaviour were strong or consistent. Specifically, the majority of the reviews indicated that there was little, limited or no evidence of effects on the behaviours studied [17, 19, 22–24]. Other reviews did not comment on the effects on behaviour [22], indicated that there may be effects in the short-term but not long-term [25], that results were “mixed” [26] or that personalised risk information may be effective, but should be judged on an individual basis and not assumed as a general principle [27].

**Table 2:** Summary of main results from each systematic review, including effect size estimates, assessment of bias, and conclusions drawn

Author (date)	Outcome (effect size)	Results of bias assessment/ Heterogeneity/ Sensitivity analyses	Conclusion
Bize (2012) [17]	Carbon monoxide testing only RR 1.06 (95%CI 0.85 to 1.32). Spirometry only RR 1.18 (95%CI 0.77 to 1.81) Spirometry + lung age RR 2.12 (95%CI 1.24 to 3.62) Carbon monoxide + spirometry – four non-significant trials – not pooled Genetic susceptibility – three non-significant trials – not pooled Carotid ultrasound – two studies, one non-significant, one significant – not pooled	13/15 trials judged to be at high or unclear risk of bias. “Only two pairs of trials were similar enough in terms of recruitment, setting and intervention to allow pooling of data for meta-analysis” (both I^2^ = 0) No sensitivity analysis	“Little evidence about the effects of most types of biomedical tests for risk assessment on smoking cessation” Only 2/15 studies showed a significant effect of the intervention on smoking cessation. “Mixed quality evidence does not support the hypothesis that other types of biomedical risk assessment increase smoking cessation in comparison to standard treatment”
DeViron (2012) [25]	Genetic notification (last follow-up) RR 1.03 (95%CI 0.64 to 1.65) Genetic notification (2 to 6 month follow up) RR 1.55 (95%CI 1.09 to 2.21)	Risk of bias apparent – only one study where patients and staff blind to intervention allocation; abstinence assessed using a variety of self-report measures. Egger’s test for publication bias non-significant (*p*=0.11 and *p*=0.76 for two follow-up periods). “No heterogeneity observed” in last follow up analysis. For 2 to 6 month follow up analysis, I^2^ = 0 “Sensitivity analysis did not identify influential studies”	Genetic notification increased smoking cessation in short term follow-up, but no evidence of long term effect.
Hackam (2012) [28]	Imaging on smoking OR 2.24 (95%CI 0.97 to 5.19). Imaging on dietary improvement OR 0.78 (95%CI 0.22 to 2.85) Imaging on physical activity (*p*=0.23)	“Most studies scored well on the methodological quality indicators”, i.e. randomization, allocation concealment, blinding, loss to follow up. For smoking analysis, I^2^ = 0	“We found limited evidence suggesting that noninvasive cardiovascular imaging alters primary prevention efforts.” Results imprecise – more research needed.
Hollands (2010) [27]	Imaging on smoking OR 2.81 (95%CI 1.23 to 6.41). Imaging on fibre consumption OR 0.36 (95%CI 0.08 to 1.53) Imaging on fat intake 1.84 (95%CI 0.51 to 6.71) Imaging on physical activity SMD 0.10 (95%CI -0.04 to 0.24)	All trials had low risk of bias. For smoking analysis, I^2^ = 0 Smoking result still significantly significant if remove one study that looked at a range of behaviours related to smoking cessation rather than cessation per se	“Due to the limited nature of the available evidence and the mixed results that were found, no strong statements can be made about the effectiveness of communicating medical imaging to change health behaviour.” “Interventions may be effective, but that this should be considered on an intervention by intervention basis, and not assumed as a general principle.”
Marteau (2010) [19]	Feedback on smoking (<6 months) OR 1.35 (95%CI 0.76 to 2.39). Feedback on smoking (>6 months) OR 1.07 (95%CI 0.64 to 1.78). Feedback on diet OR 2.24 (95%CI 1.17 to 4.27). Feedback on physical activity OR 1.03 (95%CI 0.59 to 1.80).	“Only a minority of studies could be considered to have a low risk of bias.” For smoking analysis (<6 months), I^2^ = 45%, (>6 months), I^2^ = 62% For diet analysis, I^2^ = 0%. For physical activity analysis, I^2^ = 0% Sensitivity analysis excluding one study that had incomplete reporting of missing data had little effects on overall smoking results.	“Claims that receiving DNA-based test results motivate people to change their behaviour are not supported by evidence.” “Weak evidence based on a small number of studies of limited quality.”
Rodondi (2011) [26]	No statistical aggregation of findings due to heterogeneity Feedback on smoking – 1 significant, 6 non-significant Feedback on diet – 4 significant, 2 non-significant Feedback on physical activity – 1 significant, 4 non-significant	Study quality assessed by design, methods of randomization and reporting of losses to follow-up – not blinding. Data not statistically aggregated	“Available evidence limited, with mixed results on cardiovascular risk factor control”
Sheridan (2010) [22]	No statistical aggregation of findings Risk estimates on varied smoking outcomes – 2 significant, 4 non-significant Risk estimates on diet – “mixed results” Risk estimates on physical activity – “mixed results”	2 trials judged to be good quality, 5 judged to be fair quality Data not statistically aggregated. One good quality RCT highlighted for each of three behaviours examined.	Behaviour findings reported in web-only content with little commentary. Throughout, the lack of clarity about varied education or counselling accompanying risk estimates was highlighted.
Smerecnik (2012) [24]	Genetic notification (overall analysis) OR 1.16 (95%CI 0.77 to 1.76) Genetic notification (<6 months) OR 1.87 (95%CI 1.20 to 2.92) Genetic notification (>6 months) OR 0.68 (95%CI 0.57 to 1.30)	Overall, “fair methodologies”, “somewhat questionable statistical quality”, and “poor reliability and validity”. No indication from funnel plot of publication bias. Substantial heterogeneity, I^2^ = 61% of main analysis – justifying case for analysing by short-term and long-term effects.	“Does not provide solid evidence for the proposed beneficial effects of genetic testing for smoking-related diseases on smoking cessation”.
Usher-Smith (2015) [23]	All three RCTs showed no significant effect on smoking outcomes. No statistically significant changes in the percentage who increased fruit and vegetable or fibre consumption or reduced fat at five months. No significant difference in mean accelerometer counts at one month (*p*=0.559) Two RCTs showed no difference in self-reported alcohol consumption.	Studies low, medium, and medium-high quality. Data not statistically aggregated. Differences in effect sizes “are more likely to reflect between-study heterogeneity” than variations in study quality.	“No current evidence” that providing patients with risk information changes behaviour, but “small reductions in cholesterol, blood pressure and modelled CVD are seen consistently”.

The limitations of the current evidence base were highlighted by a number of authors, in terms of number of studies but also study quality. Most reviews highlighted problems with high risk of bias in included primary studies [17, 19, 22, 24, 25], although two reviews indicated that this was not a major problem [27, 28]. Heterogeneity of study outcomes was noted in several reviews (17,19[for smoking outcomes],23,24), but a lack of heterogeneity was also reported in two reviews (19[for diet and physical activity outcomes],25).

**(b) By Nature and Source of Risk Information.** Two reviews examined the effects of communicating numerical risk information and both found little support for the idea that this results in changes in the behaviours examined [22, 23].

In the review with the largest number of primary studies, several different types of risk communication interventions were examined, but the majority of the interventions employed carbon monoxide testing or spirometry. Overall in this review only two out of 15 primary studies examining effects on smoking were statistically significant [17]. There were three groups of studies deemed by the authors to be sufficiently similar to statistically pool results, and only the studies examining the effects of communicating spirometry incorporating information about lung age were statistically significant overall (RR=2.12, 95%CI 1.24 to 3.62).

Three reviews were concerned with evaluating the effects of genetic testing [19, 24, 25]. There was total overlap in the primary studies included in two of these reviews [24, 25] which included the same five primary studies. Further, these five studies were also all included in a further genetic testing review [19], which included two further primary studies. Somewhat surprisingly given this overlap, the reviews came to rather different conclusions in relation to short-term smoking cessation. One review [25] noted a significant effect between two and six months (RR=1.55, 95%CI 1.09 to 2.21) and another [24] noted a significant effect up to six months (OR=1.87, 95%CI 1.20 to 2.92). By contrast, the third [19] did not find a significant effect up to six months (OR=1.35, 95%CI 0.76 to 2.39). None of the reviews found effects beyond 6 months, and agreed on a lack of sustained quitting, with no effects on last follow-up [25] : (RR=1.03, 95%CI 0.64 to 1.65), or effects after six months [19, 27]: (OR=1.07, 95%CI 0.64 to 1.78 and OR=0.68, 0.57 to 1.30).

The most promising method of communicating risk information appears to be those that employed imaging or visual techniques such as tomography or ultrasound, which were considered by three reviews [26–28]. In one review [27], the three trials that reported effects of visual imaging on smoking cessation were overall statistically significant (OR=2.81, 95%CI 1.23 to 6.41). However, another review found only one of seven studies to yield significant effects on smoking cessation [26]. By contrast, this latter review identified four statistically significant studies and two null studies reporting effects on diet [26]. The other review found little evidence of an effect on behaviour.

**(c) By Behaviour Targeted in Review.** All nine systematic reviews included studies on smoking. Taken as a whole, the results provided some evidence of effects of communicating personalised disease on this behaviour, with some reviews suggesting effects on cessation at least in the short-term [24, 25, 27] but other reviews finding no such support [17, 19, 22–24, 26]. There was no support for long-term smoking cessation in any review.

Six of the nine systematic reviews included studies on dietary outcomes. Taken as a whole, the results provided the best evidence of effects of communicating disease-related risks on any behavioural outcome. A review of the effects of atherosclerosis imaging feedback [26] identified four statistically significant studies and two null studies, a review of the effects of genetic testing [19] found an overall significant effect on dietary outcomes in two studies (OR=2.24, 95%CI 1.17 to 4.27) and a review of the effects of numerical coronary heart disease risk estimation [22] reported “mixed” findings in the three studies they included. By contrast, the other three reviews included only one primary study that found no significant effect of interventions on dietary outcomes [23, 27, 28] this was the same study in two of these reviews.

Six of the nine systematic reviews included studies examining physical activity. The results provided little evidence of effects on this outcome. The strongest evidence of an effect on physical activity came from a review of the effects of atherosclerosis imaging [26] that identified one statistically significant study and four statistically non-significant studies and a review of the effects of numerical coronary heart disease risk estimation [22] which reported “mixed” findings in the three studies they included. Three reviews included only one primary study each that found no significant effects on physical activity outcomes [23, 27, 28]. In line with this, a review of the effects of genetic testing [19] found an overall null effect on physical activity in two studies (OR=1.03, 95%CI 0.59 to 1.80) that was homogeneous (I^2^ = 0).

Only one systematic review included studies on alcohol consumption [23]. This review reported two studies, which both reported null effects of risk communications on alcohol consumption.

**(d) By Medical Condition for Which Risk Information Presented.** Of the nine reviews, four focussed on risk of cardio-vascular disease and specifically coronary heart disease, by investigating communication of the results of atherosclerosis imaging [26, 28], or numerical coronary heart disease risk estimates [22, 23]. One review focussed on genetic testing for cancer [24]. The other four reviews did not have a clear disease focus. There was no clear pattern of effectiveness of interventions according to which medical condition the review focussed on.

### Characteristics of Primary Studies Included in the Systematic Reviews

Characteristics of the 36 primary studies are summarised in Table 3. Details are provided for each study in Electronic Supplementary Material 3, and characteristics of primary studies included in each included systematic review are provided in Electronic Supplementary Material 4. The overall pattern of characteristics was as follows:

**(a) Addressing Self-Efficacy and Response Efficacy.** Of the 36 primary studies, only three explicitly stated that the disease risk communication interventions were targeting self-efficacy, and only one explicitly stated that the interventions were targeting response efficacy (see Table 3). Similarly, only 8 reported assessing self-efficacy and only three reported assessing response efficacy.

**(b) Behaviour Change Techniques Included in the Disease Risk Communication Interventions.** The 36 primary studies included a limited number of behaviour change techniques (see Table 3), with only ten unique behaviour change techniques found to be present out of 93 contained in the taxonomy used for coding [13]. The most commonly used behaviour change techniques were “provide information on consequences of the behaviour to the individual” (k=33) and “provide information on consequences of the behaviour in general” (k=17). The next most frequently used were “goal setting (behaviour)” (k=8) and “fear arousal (k=6).

**(c) Use of Theory in Studies.** Theory was not used much in the studies included (see Table 3). Only 10 of 36 studies explicitly mentioned a theory or model of behaviour. Similarly, only nine studies measured theory-relevant constructs or predictors, and only two studies discussed the study findings in relation to theory

**Table 3:** Frequencies of primary studies according to: (a) behaviours examined; (b) medical condition; (c) theoretical grounding of intervention; (d) use of self-efficacy and response-efficacy; (e) nature and source of risk information; and (f) behaviour change techniques

Category	Sub-category	Frequency
(a) Nature and source of risk information	Imaging/visual feedback	10
	Numerical risk estimate ^i^	9
	Carbon monoxide testing	8
	Genetic testing	7
	Spirometry	7
(b) Behaviours examined	Smoking	34
	Diet	17
	Physical activity	16
	Alcohol	5
(c) Medical condition^ii^	Coronary heart disease^iii^	19
	No condition specified/Multiple conditions specified	8
	Cancer	5
	Respiratory diseases	3
	Alzheimer’s disease	1
	Familial hypercholesterolemia	1
(d) Theoretical grounding of intervention	Theory/model of behaviour mentioned^iv^	10
	Targeted construct mentioned as predictor of behaviour	11
	Theory/predictors used to: select recipients for the intervention, develop intervention techniques, or tailor intervention techniques to recipients	12
	Theory-relevant constructs/predictors are measured	9
	At least one of the intervention techniques are explicitly linked to at least one theory-relevant construct/predictor	8
	Analysis of construct/s/predictors	8
	Results discussed in relation to theory	2
(e) Use of response-efficacy and self-efficacy	Targeted self-efficacy	3
	Targeted response-efficacy	1
	Reported self-efficacy	8
	Reported response-efficacy	3
(f) Behaviour change techniques^v^	Provide information on consequences of behaviour to the individual	33
	Provide information on consequences of behaviour in general	17
	Goal setting (behaviour)	8
	Fear arousal	6
	Motivational interviewing	4
	Stress management/Emotional control training	3
	Barrier identification/Problem solving	2
	Goal setting (outcome)	2
	Relapse prevention/ Coping planning	2
	Prompt self-monitoring of behaviour	1

^i^ One study (OXCHECK 1995) only reported information on the health check (they measured height, blood pressure and cholesterol, rather than providing overall numerical risk estimates)

^ii^ Shahab (2011) focused on cardiovascular disease and respiratory diseases

^iii^ Includes coronary heart disease and atherosclerosis

^iv^ Studies were coded as ‘no’ if theory was only explained to participants in the methods, rather than mentioning the theory and the relations among variables

^V^ In one study (Jamrozik, 1984), the health visitor intervention group was excluded as this is not relevant to risk information studies

.

## Discussion

Across a broad range of methods of assessing and communicating risk information, the present review of reviews found little evidence that personalised risk information had strong or consistent effects on health-related behaviours. The most promising effects came from reviews of imaging/visual risk feedback and effects on smoking and dietary behaviours, although with little evidence of sustained change and with more null findings than significant ones. Effects of providing numerical risk information and effects on physical activity were particularly unpromising. The primary studies included in these interventions appeared to be mainly atheoretical, with little targeting of response efficacy or self-efficacy, factors that are known to augment the impact of risk information on behaviour.

Overall, the effects of personalised risk information on the four behaviours examined were not consistent and where changes were observed, they were not maintained. The quality of the reviews was judged to be good, although most of the review authors criticised the quality of many of the primary studies they included. Most reviews noted that the primary literature they covered was limited not only in quality of studies, but also in number of primary studies, making it difficult to draw definitive conclusions.

The forms of risk provision that were most promising were those that used visual/imaging approaches to communicate risk information [26–28]. By contrast, studies involving the provision of numerical risk information were least promising [22, 23]. These findings are consistent with the broader literature on risk communication [29], which highlights the importance of how imagery can be more strongly associated with behaviour as it can more strongly influence automatic processes compared to numerical statements that are difficult to evaluate [30].

Most primary studies were concerned with smoking, and significant effects on behaviour were found for studies that provided medical imaging [27], spirometry plus information about lung age [17], and possibly genetic testing, but only in the short term [19, 24, 25]. By contrast, there was little evidence of smoking cessation brought about by other methods of personalised risk communication [17, 22, 23, 26, 28]. Further, all studies of genetic testing agreed there were limited effects of genetic testing on smoking behaviour in the longer term. Taking all these findings into account, a reasonable conclusion would be that although some forms of communicating personalised disease-related risks may influence smoking behaviours, there is no evidence that these effects are strong or consistent.

There was also some evidence of the effects of the communicating disease-related risks on dietary outcomes, with three of the reviews finding support or mixed support for effects on dietary outcomes, and the other three reviews finding no support based on only two studies. This pattern contrasts with studies of physical activity with two reviews finding mixed support for effects on this outcome and the other four reviews finding no support. One possible explanation for this discrepancy may be that people’s mental models may find it more sensible to address physiological markers such as artery atherosclerosis through reducing dietary fat than through increasing physical activity. Such an observation has been made in the context of diabetes, where patients more easily see the benefits of changing diet to tackle blood glucose than increasing physical activity [31]. Another explanation for this discrepancy is that objective measures of physical activity are far easier to use than those for diet, and that self-report measures of physical activity are more valid than those of diet [32, 33]. Hence, given the lack of blinding in many primary studies, the results may be more likely to be biased away from the null for dietary outcomes, which were assessed using unvalidated self-report measures in the primary studies included.

These overall findings fit well with the broader literature on disease risk communication, which suggested an overall small effect (d=0.23) on behaviour when risk appraisals are increased [9]. Personalised risk communication might be expected to lead to greater understanding than general non personal risk communication [18]. Further, personalised risk communications should lead to greater acceptance of the message regarding risk [34]. Together, these two processes might suggest that personalised risk communications should be successful at increasing risk appraisals and thereby behaviour [34]. Despite this, there was no good evidence from these reviews that effects on behaviour were any stronger than non-personalised communications. One plausible reason for the generally limited effects of these personalised risk communications is that they did not generally target self-efficacy and response efficacy, which would be likely to increase the impact of risk information on health-related behaviours [8–10].

The present systematic review of reviews has many strengths. First it follows an established method of conducting such a systematic review of systematic reviews, with explicit procedures for the selection, appraisal and synthesis of individual systematic reviews [15]. Second, it brings together a diverse set of systematic reviews and thereby allows the commonalities and differences in the findings of these reviews to be highlighted. In particular, although there are some areas that appear promising, the present review has shown that across a range of different methods of personalised risk communication, it seems highly unlikely that providing personalised risk information will have strong or consistent effects on health-related behaviour. Further, by quantifying aspects of primary studies, such as use of theory and behaviour change technique content of interventions, we have shown that a plausible reason for the lack of effectiveness of the personalised risk interventions is that they do not utilise knowledge from the wider literature on risk communication.

The nine systematic reviews that are included in the present systematic review of reviews were generally well-conducted, with AMSTAR scores of 9 or ten out of eleven for most of the reviews. This review quality is encouraging, given that a recent systematic survey of systematic reviews found many examples of poor quality, including at least a third omitting descriptions of search strategies or methods of quality appraisal [35]. By contrast, the quality of the primary studies included in these systematic reviews were often judged to be poor, a finding that was raised in the main conclusions of the systematic reviews (and extracted into Table 2).

The main implication for practice of the findings presented is that one can now be reasonably confident that communicating personalised risk information on its own is unlikely to lead to much sustained behaviour change, irrespective of the nature and source of information. Greater targeting of response efficacy and self-efficacy than was evident in the studies included in these systematic reviews may result in greater changes in these health-related behaviours. It is important to note however, that there is now strong evidence that failure to change health-related behaviour is often not due to insufficient motivation. Instead, failure to change behaviour is more often due to motivated people lacking the skills to self-regulate their own behaviour [36]. Successful behaviour change interventions are therefore likely to involve behaviour change techniques promoting more effective self-regulation e.g. planning and self-monitoring on behaviour for which there is a stronger evidence base than for risk communications [12]. These behaviour change techniques were not included in the primary studies included in the systematic reviews that we considered.

In terms of future research, there is a need for more primary studies of better quality, and particularly with better measures of diet and physical activity. Given that the most promising studies have used imaging techniques, it would seem sensible for evaluations of these sources of risk estimation to be prioritised. Future studies should aim to look at maintenance of behaviour change as well as behaviour initiation. More comparisons of personalised versus non-personalised risk communications would be useful, given the dearth of such studies. These studies could also usefully compare the effects of personalised risk communication to the effects on non-personalised risk communication, when both are used in conjunction with more evidence-based intervention behaviour change techniques, to promote changes in self-efficacy, response efficacy and self-regulation.

It may be that personalised risk communications may be best suited to motivating people to engage in effective behaviour change programmes, by motivating attempts to change behaviour [30]. Given this, it would be useful to compare these risk communication strategies in terms of whether they promote uptake of evidence-based behaviour change programmes, since it appears unlikely that sustained behaviour change will be brought about solely by communicating personalised risk.

We believe that the present systematic review of systematic reviews has provided a clearer picture of the effects of communicating personalised risk information on health-related behaviour, with two key messages. Firstly, that the literature on personalised risk communication would benefit from greater consideration of the theoretical and empirical literatures on general risk communication. Secondly, presenting risk information on its own, even when highly personalised, does not produce strong effects on lifestyle behaviours or changes which are sustained. Future research should therefore consider how best to use personalised risk information to engage people in behaviour change programmes that are more likely to be effective in producing changes in behaviour.

### Electronic supplementary material

ESM 1 (DOCX 35 kb)

## Appendix 1

### Search terms used for MEDLINE database

1. exp. Risk/
2. exp. Disease Susceptibility/.
3. ((tailor* or personal* or individual*) adj2 (counsel* or message* or material* or intervention*)).tw.
4. (tailored or tailoring or individuali*ed or peronali*ed).tw.
5. (risk* or susceptib*).tw.
6. or/1–5.
7. communication/.
8. persuasive communication/.
9. counselling/.
10. genetic counselling/.
11. health promotion/.
12. health education/.
13. Patient Education as Topic/.
14. Health Knowledge, Attitudes, Practice/.
15. attitude to health/.
16. patient acceptance of healthcare/.
17. or/7–16.
18. 6 and 17.
19. (risk* adj3 (notif* or inform* or communicat* or counsel* or apprais* or assess* or perception* or perceiv*)).tw.
20. 18 or 19.
21. (diet* or smok* or tobacco or alcohol or weight or “physical* activ*” or exerci* or lifestyle*).tw.
22. 20 and 21.
23. limit 22 to (english language and humans and yr.=“2008 -Current” and systematic reviews)
