# Supplementary material for: Can Communicating Personalised Disease Risk Promote Healthy Behaviour Change? A Systematic Review of Systematic Reviews
Source: Ann Behav Med. 2017 Mar 13;51(5):718–29. doi: 10.1007/s12160-017-9895-z (PMC5602036; doi:10.1007/s12160-017-9895-z)
Supplement: Supplementary file 1 — (DOCX 35 kb) [file 12160_2017_9895_MOESM1_ESM.docx]

**Electronic Supplementary Material 1: Papers excluded at the full-text screening stage**

Bach, P.B., Mirkin, J.N., Oliver, T.K., Azzoli, C.G., Berry, D.A., Brawley, O.W., Byers, T., Colditz, G.A., Gould, M.K., Jett, J.R. Sabichi, A.L. (2012). Benefits and harms of CT screening for lung cancer: a systematic review. *Journal of the American Medical Association*, 307(22): 2418-2429.

Bize, R., Burnand, B., Mueller, Y., Rège Walther, M., Cornuz, J. (2009). Biomedical risk assessment as an aid for smoking cessation. *Cochrane Database of Systematic Reviews*, (2): CD004705.

De Viron, S., Malats, N., Van der Heyden, J., Van Oyen, H., Brand, A. (2013). Environmental and genomic factors as well as interventions influencing smoking cessation: a systematic review of reviews and a proposed working model. *Public Health Genomics*, 16(4): 159-173.

Heshka, J. T., Palleschi, C., Howley, H., Wilson, B., Wells, P. S. (2008). A systematic review of perceived risks, psychological and behavioral impacts of genetic testing. *Genetics in Medicine*, 10(1): 19-32.

Hilgart, J. S., Coles, B., Iredale, R. (2012). Cancer genetic risk assessment for individuals at risk of familial breast cancer. *Cochrane Database of Systematic Reviews*, (2): CD003721.

Lin, K., Watkins, B., Johnson, T., Rodriguez, J. A., Barton, M. B. (2008). Screening for chronic obstructive pulmonary disease using spirometry: summary of the evidence for the US Preventive Services Task Force. *Annals of Internal Medicine*, 148(7): 535-543.

Mamudu, H. M., Paul, T. K., Veeranki, S. P., Budoff, M. (2014). The effects of coronary artery calcium screening on behavioral modification, risk perception, and medication adherence among asymptomatic adults: a systematic review. *Atherosclerosis*, 236(2): 338-350.

Palomaki, G. E., Melillo, S., Neveux, L., Douglas, M. P., Dotson, W. D., Janssens, A. C. J., Balkite, E.A., Bradley, L. A. (2010). Use of genomic profiling to assess risk for cardiovascular disease and identify individualized prevention strategies—a targeted evidence-based review. *Genetics in Medicine*, 12(12): 772-784.

Poghosyan, H., Sheldon, L. K., Cooley, M. E. (2012). The impact of computed tomography screening for lung cancer on smoking behaviors: a teachable moment?. *Cancer Nursing*, 35(6): 466-475.

Roberts, J. S., Ostergren, J. (2013). Direct-to-consumer genetic testing and personal genomics services: a review of recent empirical studies. *Current Genetic Medicine Reports*, 1(3): 182-200.

Scheuner, M. T., Sieverding, P., Shekelle, P. G. (2008). Delivery of genomic medicine for common chronic adult diseases: a systematic review. *Journal of the American Medical Association*, 299(11): 1320-1334.

Schneider, K. I., Schmidtke, J. (2014). Patient compliance based on genetic medicine: a literature review. *Journal of Community Genetics*, 5(1): 31-48.

Senore, C., Giordano, L., Bellisario, C., Di Stefano, F., Segnan, N. (2012). Population based cancer screening programmes as a teachable moment for primary prevention interventions. A review of the literature. *Frontiers in Oncology*, 2: 45.

Sheridan, S. L., Crespo, E. (2008). Does the routine use of global coronary heart disease risk scores translate into clinical benefits or harms? A systematic review of the literature. *BMC Health Services Research*, 8(1): 1.

Slatore, C.G., Baumann, C., Pappas, M., Humphrey, L. L. (2014). Smoking behaviors among patients receiving computed tomography for lung cancer screening. Systematic review in support of the US Preventive Services Task Force. *Annals of the American Thoracic Society*, 11(4): 619-627.

Vernarelli, J. A. (2013). Impact of genetic risk assessment on nutrition-related lifestyle behaviours. *Proceedings of the Nutrition Society*, 72(01): 153-159.

Waldron, C. A., van der Weijden, T., Ludt, S., Gallacher, J., Elwyn, G. (2011). What are effective strategies to communicate cardiovascular risk information to patients? A systematic review. *Patient Education and Counseling*, 82(2): 169-181.

Welton, N. J., Johnstone, E. C., David, S. P., Munafò, M. R. (2008). A cost-effectiveness analysis of genetic testing of the DRD2 Taq1A polymorphism to aid treatment choice for smoking cessation. *Nicotine & Tobacco Research*, 10(1): 231-240.

Whelton, S. P., Nasir, K., Blaha, M. J., Gransar, H., Metkus, T. S., Coresh, J., Berman, D.S., Blumenthal, R. S. (2012). Coronary artery calcium and primary prevention risk assessment: what is the evidence? An updated meta-analysis on patient and physician behavior. *Circulation: Cardiovascular Quality and Outcomes*, 5(4): 601-607.

**Electronic Supplementary Material 2: Summary of quality ratings (AMSTAR scores) for each included systematic review**

|  | **Bize**  **(2012)** | **DeViron (2012)** | **Hackam (2012)** | **Hollands (2010)** | **Marteau (2010)** | **Rodondi (2011)** | **Sheridan (2010)** | **Smerecnik (2012)** | **Usher-Smith (2015)** |
| --- | --- | --- | --- | --- | --- | --- | --- | --- | --- |
| 1. Was an a priori design included? | Yes | Yes | Yes | Yes | Yes | Yes | Yes | Yes | Yes |
| 2. Was there duplicate study selection and data extraction? | Yes | Yes | Yes | Yes | Yes | Yes | Yes | Yes | Yes |
| 3. Was a comprehensive literature search performed? | Yes | Yes | Yes | Yes | Yes | Yes | Yes | Yes | Yes |
| 4. Was the status of publication (i.e. grey literature) used as an inclusion criterion? | Yes | Yes | Yes | Yes | Yes | No | No | Yes | Yes |
| 5. Was a list of studies (included and excluded) provided? | Yes | No | No | Yes | Yes | No | No | No | No |
| 6. Were the characteristics of the included studies provided? | Yes | Yes | Yes | Yes | Yes | Yes | Yes | Yes | Yes |
| 7. Was the scientific quality of the included studies assessed and documented? | Yes | Yes | Yes | Yes | Yes | Yes | Yes | Yes | Yes |
| 8. Was the scientific quality of the included studies used appropriately in formulating conclusions? | Yes | Yes | Yes | Yes | Yes | Cannot answer | Yes | Yes | Yes |
| 9. Were the methods used to combine the findings of studies appropriate? | Yes | Yes | Yes | Yes | Yes | Yes | Yes | Yes | Yes |
| 10. Was the likelihood of publication bias assessed? | No | No | No | n/a | Yes | No | No | Yes | No |
| 11. Was the conflict of interest included? | Yes | Yes | Yes | Yes | Yes | Yes | Yes | Yes | Yes |
| **TOTAL AMSTAR SCORE** | 10 | 9 | 9 | 10 | 11 | 7 | 8 | 10 | 9 |

**Electronic Supplementary Material 3: Summary table showing the following characteristics for each primary study: (a) behaviors examined; (b) medical condition; (c) theoretical grounding of intervention; (d) use of self-efficacy and response-efficacy; (e) nature and source of risk information; and (f) behavior change techniques**

| **Author (reference)** | **Nature and source of risk information** | **Behaviors examined** | **Medical conditioni** | **Theoretical grounding of interventionii** | **Use of response-efficacy and self-efficacy** | **Behavior change techniques** |
| --- | --- | --- | --- | --- | --- | --- |
| **Audrain et al. [1,2]** | Carbon monoxide testing; Genetic testing | Smoking | Cancer | Theory/model of behavior mentioned;  Targeted construct mentioned as predictor of behavior;  Theory/predictors used to: select recipients for the intervention, develop intervention techniques, or tailor intervention techniques to recipients;  At least one of the intervention techniques are explicitly linked to at least one theory-relevant construct/predictor;  Theory-relevant constructs/predictors are measured;  Analysis of construct/s/predictors;  Results discussed in relation to theory | Reported self-efficacy;  Reported response-efficacy | Goal setting (behavior); Provide information on consequences of behavior to the individual;  Provide information on consequences of behavior in general |
| **Bovet et al. [3,4]** | Imaging/visual feedback | Smoking | Cardiovascular disease | - | - | Fear arousal;  Provide information on consequences of behavior in general |
| **Buffels et al. [5]** | Spirometry | Smoking | Respiratory diseases | - | - | Goal setting (behavior); Motivational interviewing |
| **Chao et al. [6]** | Genetic testing | Diet; Physical activity | Alzheimer's disease | - | - | Provide information on consequences of behavior to the individual |
| **Edelman et al. [7]iii** | Numerical risk estimate | Diet; Physical activity | Cardiovascular disease | - | - | Barrier identification/Problem solving;  Goal setting (behavior); Provide information on consequences of behavior in genera;  Provide information on consequences of behavior in general to the individual;  Relapse prevention/ Coping planning;  Stress management/Emotional control training |
| **Family Heart Study Group [8]** | Numerical risk estimate | Smoking; Alcohol; Diet; Physical activity | Cardiovascular disease | - | - | Provide information on consequences of behavior to the individual |
| **Hanlon et al. [9]** | Numerical risk estimate | Smoking; Diet; Physical activity | Cardiovascular disease | - | - | Provide information on consequences of behavior in general;  Provide information on consequences of behavior to the individual |
| **Hishida et al. [10]** | Genetic testing | Smoking | Cancer | Theory/model of behavior mentioned;  Targeted construct mentioned as predictor of behavior;  Theory/predictors used to: select recipients for the intervention, develop intervention techniques, or tailor intervention techniques to recipients | - | Provide information on consequences of behavior in general;  Provide information on consequences of behavior to the individual |
| **Ito et al. [11]** | Genetic testing | Smoking | Cancer | Theory/model of behavior mentioned | - | Provide information on consequences of behavior to the individual |
| **Jamrozik et al. [12]iv** | Carbon monoxide testing | Smoking | No condition specified/Multiple conditions specified | - | - | Provide information on consequences of behavior in general;  Provide information on consequences of behavior to the individual |
| **Kalia et al. [13]** | Imaging/visual feedback | Smoking; Diet; Physical activity | Cardiovascular disease | Theory/model of behavior mentioned | - | Fear arousal; Provide information on consequences of behavior to the individual |
| **Lederman et al. [14]** | Imaging/visual feedback | Smoking; Diet; Physical activity | Cardiovascular disease | - | - | Fear arousal;  Provide information on consequences of behavior to the individual |
| **Lovibond et al. [15]** | Numerical risk estimate | Smoking; Diet; Physical activity | Cardiovascular disease | Targeted construct mentioned as predictor of behavior;  Theory/predictors used to: select recipients for the intervention, develop intervention techniques, or tailor intervention techniques to recipients;  At least one of the intervention techniques are explicitly linked to at least one theory-relevant construct/predictor | Reported self-efficacy | Barrier identification/Problem solving;  Goal setting (behavior); Prompt self-monitoring of behavior;  Provide information on consequences of behavior in general;  Provide information on consequences of behavior to the individual;  Stress management/Emotional control training |
| **Lowensteyn et al. [16]** | Numerical risk estimate | Smoking | Cardiovascular disease | - | - | Provide information on consequences of behavior to the individual |
| **Marteau et al. [17]** | Genetic testing | Smoking; Diet; Physical activity | Familial hypercholesterolemi-a | Targeted construct mentioned as predictor of behavior;  Theory/predictors used to: select recipients for the intervention, develop intervention techniques, or tailor intervention techniques to recipients;  At least one of the intervention techniques are explicitly linked to at least one theory-relevant construct/predictor;  Theory-relevant constructs/predictors are measured;  Analysis of construct/s/predictors | Reported self-efficacy;  Reported response-efficacy;  Targeted self-efficacy;  Targeted response-efficacy | Provide information on consequences of behavior to the individual |
| **McBride et al. [18]** | Genetic testing | Smoking | Cancer | Theory/model of behavior mentioned;  Theory/predictors used to: select recipients for the intervention, develop intervention techniques, or tailor intervention techniques to recipients | - | Motivational interviewing; Provide information on consequences of behavior in general;  Provide information on consequences of behavior to the individual |
| **McClure et al. [19,20]** | Carbon monoxide testing; Spirometry | Smoking | No condition specified/Multiple conditions specified | Theory/model of behavior mentioned;  Targeted construct mentioned as predictor of behavior;  Theory/predictors used to: select recipients for the intervention, develop intervention techniques, or tailor intervention techniques to recipients;  At least one of the intervention techniques are explicitly linked to at least one theory-relevant construct/predictor;  Theory-relevant constructs/predictors are measured;  Analysis of construct/s/predictors | Reported self-efficacy | Motivational interviewing; Provide information on consequences of behavior in general;  Provide information on consequences of behavior to the individual |
| **Obuchowski et al. [21]** | Imaging/visual feedback | Smoking; Alcohol; Diet; Physical activity | No condition specified/Multiple conditions specified | - | - | Provide information on consequences of behavior to the individual |
| **O'Malley [22]** | Imaging/visual feedback | Smoking | Cardiovascular disease | Targeted construct mentioned as predictor of behavior;  Theory/predictors used to: select recipients for the intervention, develop intervention techniques, or tailor intervention techniques to recipients;  Theory-relevant constructs/predictors are measured;  Analysis of construct/s/predictors | - | Provide information on consequences of behavior to the individual |
| **O'Malley et al. [23,24]** | Imaging/visual feedback | Smoking; Diet; Physical activity | Cardiovascular disease | Targeted construct mentioned as predictor of behavior;  Theory/predictors used to: select recipients for the intervention, develop intervention techniques, or tailor intervention techniques to recipients;  At least one of the intervention techniques are explicitly linked to at least one theory-relevant construct/predictor | - | Fear arousal;  Provide information on consequences of behavior to the individual |
| **Orakzai et al. [25]** | Imaging/visual feedback | Smoking; Diet; Physical activity | Cardiovascular disease | - | - | Fear arousal |
| **OXCHECK Study Group [26,27]** | Numerical risk estimatev | Smoking; Alcohol; Diet; Physical activity | Cardiovascular disease | - | - | Provide information on consequences of behavior to the individual |
| **Parkes et al. [28,29]** | Spirometry | Smoking | Respiratory diseases | - | - | Provide information on consequences of behavior to the individual |
| **Price et al. [30]** | Numerical risk estimate | Smoking; Alcohol; Diet; Physical activity | Cardiovascular disease | Theory/model of behavior mentioned;  Theory/predictors used to: select recipients for the intervention, develop intervention techniques, or tailor intervention techniques to recipients; Theory-relevant constructs/predictors are measured;  Analysis of construct/s/predictors; | - | Barrier identification/Problem solving; Goal setting (behavior);  Goal setting (outcome); Provide information on consequences of behavior in general;  Provide information on consequences of behavior to the individual; |
| **Risser et al. [31]** | Carbon monoxide testing; Spirometry | Smoking | No condition specified/Multiple conditions specified | - | - | Goal setting (behavior); Provide information on consequences of behavior in general;  Provide information on consequences of behavior to the individual |
| **Rodondi et al. [32,33]** | Imaging/visual feedback | Smoking | Cardiovascular disease | Targeted construct mentioned as predictor of behavior;  Theory-relevant constructs/predictors are measured | - | Fear arousal;  Goal setting (behavior); Provide information on consequences of behavior in general;  Provide information on consequences of behavior to the individual |
| **Sanders et al. [34]** | Carbon monoxide testing | Smoking | No condition specified/Multiple conditions specified | - | - | Provide information on consequences of behavior in general;  Provide information on consequences of behavior to the individual |
| **Sanderson et al. [35]** | Genetic testing | Smoking | Cancer | - | Reported self-efficacy | Provide information on consequences of behavior in general;  Provide information on consequences of behavior to the individual |
| **Sandwell et al. [36]** | Imaging/visual feedback | Smoking; Diet; Physical activity | Cardiovascular disease | - | - | Provide information on consequences of behavior to the individual |
| **Segnan et al. [37]** | Spirometry | Smoking | No condition specified/Multiple conditions specified | - | - | Provide information on consequences of behavior to the individual |
| **Shahab et al. [38]** | Carbon monoxide testing | Smoking | Cardiovascular disease; Respiratory diseases | Theory/model of behavior mentioned;  Targeted construct mentioned as predictor of behavior;  Theory/predictors used to: select recipients for the intervention, develop intervention techniques, or tailor intervention techniques to recipients;  At least one of the intervention techniques are explicitly linked to at least one theory-relevant construct/predictor;  Theory-relevant constructs/predictors are measured;  Analysis of construct/s/predictors;  Results discussed in relation to theory | Reported self-efficacy;  Reported response-efficacy;  Targeted self-efficacy | Provide information on consequences of behavior in general;  Provide information on consequences of behavior to the individual |
| **Sippel et al. [39]** | Carbon monoxide testing; Spirometry | Smoking | No condition specified/Multiple conditions specified | - | - | Provide information on consequences of behavior in general;  Provide information on consequences of behavior to the individual |
| **Walker et al. [40]** | Carbon monoxide testing; Spirometry | Smoking | No condition specified/Multiple conditions specified | Targeted construct mentioned as predictor of behavior;  Theory/predictors used to: select recipients for the intervention, develop intervention techniques, or tailor intervention techniques to recipients;  Theory-relevant constructs/predictors are measured;  Analysis of construct/s/predictors | Reported self-efficacy | Provide information on consequences of behavior to the individual;  Relapse prevention/Coping planning;  Stress management/Emotional control training |
| **Williams et al. [41]** | Numerical risk estimate | Smoking; Diet | Cardiovascular disease | Theory/model of behavior mentioned;  Targeted construct mentioned as predictor of behavior;  Theory/predictors used to: select recipients for the intervention, develop intervention techniques, or tailor intervention techniques to recipients;  At least one of the intervention techniques are explicitly linked to at least one theory-relevant construct/predictor;  Theory-relevant constructs/predictors are measured;  Analysis of construct/s/predictors;  Results discussed in relation to theory | Reported self-efficacy;  Targeted self-efficacy | Provide information on consequences of behavior in general;  Provide information on consequences of behavior to the individual |
| **Wister et al. [42]** | Numerical risk estimate | Smoking; Diet; Physical activity | Cardiovascular disease | Theory/model of behavior mentioned;  At least one of the intervention techniques are explicitly linked to at least one theory-relevant construct/predictor | - | Goal setting (behavior);  Goal setting (outcome); Motivational interviewing; Provide information on consequences of behavior to the individual |
| **Wong et al. [43]** | Imaging/visual feedback | Smoking; Diet; Physical activity | Cardiovascular disease | - | - | Provide information on consequences of behavior to the individual |

i Cardiovascular disease includes coronary heart disease and atherosclerosis

ii For the question ‘Theory/model of behavior mentioned’, studies were coded as ‘no’ if theory was only explained to participants in the methods, rather than mentioning the theory and the relations among variables

iii Smoking was not addressed by the researchers owing to the small number of smokers in the study

iv The health visitor intervention group in this study was excluded as it is not relevant to risk information studies

v This study only reported information on the health check (they measured height, blood pressure and cholesterol), rather than providing overall numerical risk estimates

**References for Electronic Supplementary Material 3.**

1. Audrain J, Boyd NR, Roth J, Main D, Caporaso NE, Lerman C. Genetic susceptibility testing in smoking-cessation treatment: one-year outcomes of a randomized trial. Addict Behav 1997; 22(6): 741-751.
2. Lerman C, Gold K, Audrain J, Lin TH, Boyd NR, Orleans CT, Wilfond B, Louben G, Caporaso N. Incorporating biomarkers of exposure and genetic susceptibility into smoking cessation treatment: effects on smoking-related cognitions, emotions, and behavior change. Health Psychology 1997; 16(1): 87-99.
3. Bovet P, Perret F, Cornuz J, Quilindo J, Paccaud F. Improved smoking cessation in smokers given ultrasound photographs of their own atherosclerotic plaques. Prev Med 2002; 34(2):215-220.
4. Bovet P, Perret F, Shamlaye C, Darioli R, Paccaud F. The Seychelles Heart Study II: methods and basic findings. Seychelles Medical & Dental Journal 1997; 5: 8-24.
5. Buffels J, Degryse J, Decramer M, Heyrman J. Spirometry and smoking cessation advice in general practice: a randomised clinical trial. Resp Med 2006; 100(11): 2012-2017.
6. Chao S, Roberts JS, Marteau TM, Silliman R, Cupples LA, Green RC. Health behavior changes after genetic risk assessment for Alzheimer disease: The REVEAL Study. Alz Dis Assoc Dis 2008; 22(1): 94-97.
7. Edelman D, Oddone EZ, Liebowitz RS, Yancy WS, Olsen MK, Jeffreys AS, Moon SD, Harris AC, Smith LL, Quillian‐Wolever RE, Gaudet TW. A multidimensional integrative medicine intervention to improve cardiovascular risk. J G Intern Med 2006; 21(7):728-734.
8. Family Heart Study Group. Randomised controlled trial evaluating cardiovascular screening and intervention in general practice: principal results of British family heart study. BMJ 1994; 308(6924): 313-320.
9. Hanlon P, McEwen J, Carey L, Gilmour H, Tannahill C, Tannahill A, Kelly M. Health checks and coronary risk: further evidence from a randomised controlled trial. BMJ 1995; 311(7020): 1609-1613.
10. Hishida A, Terazawa T, Mamiya T, Ito H, Matsuo K, Tajima K, Hamajima N. Efficacy of genotype notification to Japanese smokers on smoking cessation—an intervention study at workplace. Cancer Epidemiol 2010; 34(1): 96-100.
11. Ito H, Matsuo K, Wakai K, Saito T, Kumimoto H, Okuma K, Tajima K, Hamajima N. An intervention study of smoking cessation with feedback on genetic cancer susceptibility in Japan. Prev Med 2006; 42(2): 102-108.
12. Jamrozik K, Vessey M, Fowler G, Wald N, Parker G, Van Vunakis H. Controlled trial of three different antismoking interventions in general practice. Br Med J (Clin Res Ed) 1984; 288(6429): 1499-503.
13. Kalia NK, Miller LG, Nasir K, Blumenthal RS, Agrawal N, Budoff MJ. Visualizing coronary calcium is associated with improvements in adherence to statin therapy. Atherosclerosis 2006; 185(2): 394-399.
14. Lederman J, Ballard J, Njike VY, Margolies L, Katz DL. Information given to postmenopausal women on coronary computed tomography may influence cardiac risk reduction efforts. J Clin Epidemiol 2007; 60(4): 389-396.
15. Lovibond SH, Birrell PC, Langeluddecke P. Changing coronary heart disease risk-factor status: the effects of three behavioral programs. J Behav Med 1986; 9(5): 415-437.
16. Lowensteyn I, Joseph L, Levinton C, Abrahamowicz M, Steinert Y, Grover S. Can computerized risk profiles help patients improve their coronary risk? The results of the Coronary Health Assessment Study (CHAS). Prev Med 1998; 27(5): 730-737.
17. Marteau T, Senior V, Humphries SE, Bobrow M, Cranston T, Crook MA, Day L, Fernandez M, Horne R, Iversen A, Jackson Z. Psychological impact of genetic testing for familial hypercholesterolemia within a previously aware population: a randomized controlled trial. Am J Med Genet Pt A 2004; 128(3): 285-293.
18. McBride CM, Bepler G, Lipkus IM, Lyna P, Samsa G, Albright J, Datta S, Rimer BK. Incorporating genetic susceptibility feedback into a smoking cessation program for African-American smokers with low income. Cancer Epidemiol Biomarkers & Prev 2002; 11(6): 521-528.
19. McClure JB, Ludman E, Grothaus L, Pabiniak C, Richards J, Mohelnitzky A. Immediate and short-term impact of a brief motivational smoking intervention using a biomedical risk assessment: The Get PHIT trial. Nicotine & Tobacco Res 2009; 11(4): 394-403.
20. McClure JB, Ludman EJ, Grothaus L, Pabiniak C, Richards J. Impact of a brief motivational smoking cessation intervention: the Get PHIT randomized controlled trial. Am J Prev Med. 2009; 37(2): 116-123.
21. Obuchowski NA, Holden D, Modic MT, Cheah G, Fu AZ, Brant-Zawadzki M, Seballos R, Mohammed TL. Total-body screening: preliminary results of a pilot randomized controlled trial. J Am Coll Radiol 2007; 4(9): 604-611.
22. O'Malley PG. Does the diagnosis of coronary calcification with electron beam computed tomography motivated behavioral change in smokers?. Military Med 2002; 167(3): 211-214.
23. O'Malley PG, Feuerstein IM, Taylor AJ. Impact of electron beam tomography, with or without case management, on motivation, behavioral change, and cardiovascular risk profile: a randomized controlled trial. JAMA 2003; 289(17): 2215-2223.
24. O’Malley PG, Taylor AJ, Gibbons RV, Feuerstein IM, Jones DL, Vernalis M, Brazaitis M. Rationale and design of the Prospective Army Coronary Calcium (PACC) Study: utility of electron beam computed tomography as a screening test for coronary artery disease and as an intervention for risk factor modification among young, asymptomatic, active-duty United States Army Personnel. Am Heart J 1999; 137(5): 932-941.
25. Orakzai RH, Nasir K, Orakzai SH, Kalia N, Gopal A, Musunuru K, Blumenthal RS, Budoff MJ. Effect of patient visualization of coronary calcium by electron beam computed tomography on changes in beneficial lifestyle behaviors. Am J Cardiol 2008; 101(7): 999-1002.
26. OXCHECK Study Group. Effectiveness of health checks conducted by nurses in primary care: final results of the OXCHECK study. BMJ 1995: 1099-1104.
27. Muir J, Mant D, Jones L, Yudkin P. Effectiveness of health checks conducted by nurses in primary care: results of the OXCHECK study after one year. BMJ 1994; 308(6924): 308-312.
28. Parkes G, Greenhalgh T, Griffin M, Dent R. Effect on smoking quit rate of telling patients their lung age: the Step2quit randomised controlled trial. BMJ 2008; 336(7644): 598-600.
29. Deane K, Stevermer JJ, Hickner J. Help smokers quit: Tell them their “lung age”. Priority Updates to Research Literature (PURLs). 2008.
30. Price HC, Griffin SJ, Holman RR. Impact of personalized cardiovascular disease risk estimates on physical activity—a randomized controlled trial. Diabetic Med 2011; 28(3): 363-372.
31. Risser MN, Belcher DW. Adding spirometry, carbon monoxide, and pulmonary symptom results to smoking cessation counseling. J G Int Med 1990; 5(1): 16-22.
32. Rodondi N, Bovet P, Hayoz D, Cornuz J. The Impact of CAROtid plaque Screening on Smoking (CAROSS) cessation and control of other cardiovascular risk factors: rationale and design of a randomized controlled trial. Contemp Clin Trials 2008; 29(5): 767-773.
33. Rodondi N, Collet TH, Nanchen D, Locatelli I, Depairon M, Aujesky D, Bovet P, Cornuz J. Impact of carotid plaque screening on smoking cessation and other cardiovascular risk factors: a randomized controlled trial. Arch Intern Med 2012; 172(4): 344-352.’
34. Sanders D, Fowler G, Mant D, Fuller A, Jones L, Marzillier J. Randomized controlled trial of anti-smoking advice by nurses in general practice. J R Coll Gen Pract 1989; 39(324): 273-276.
35. Sanderson SC, Humphries SE, Hubbart C, Hughes E, Jarvis MJ, Wardle J. Psychological and Behavioural Impact of Genetic Testing Smokers for Lung Cancer Risk A Phase II Exploratory Trial. J Health Psychol. 2008 May 1;13(4):481-94.
36. Sandwell JC, Wingard DL, Laughlin GA, Barrett‐Connor E. Electron beam computed tomography screening and heart disease risk factor modification. Prev Cardiol 2006; 9(3): 133-7.
37. Segnan N, Ponti A, Battista RN, Senore C, Rosso S, Shapiro SH, Aimar D. A randomized trial of smoking cessation interventions in general practice in Italy. Canc Causes & Control 1991; 2(4): 239-246.
38. Shahab L, West R, McNeill A. A randomised controlled trial of adding expired air-carbon-monoxide feedback to brief stop smoking advice: evaluation of cognitive and behavioral effects. Health Psychol 2011; 30: 49-57.
39. Sippel JM, Osborne ML, Bjornson W, Goldberg B, Buist AS. Smoking cessation in primary care clinics. J G Intern Med 1999; 14(11): 670-676.
40. Walker WB, Franzini LR. Low-risk aversive group treatments, physiological feedback, and booster sessions for smoking cessation. Behav Ther 1985; 16(3): 263-274.
41. Williams GC, McGregor H, Sharp D, Kouides RW, Lévesque CS, Ryan RM, Deci EL. A Self‐Determination Multiple Risk Intervention Trial to Improve Smokers' Health. J G Intern Med 2006; 21(12): 1288-1294.
42. Wister A, Loewen N, Kennedy-Symonds H, McGowan B, McCoy B, Singer J. One-year follow-up of a therapeutic lifestyle intervention targeting cardiovascular disease risk. Canadian Med Assoc J 2007; 177(8): 859-865.
43. Wong ND, Detrano RC, Diamond G, Rezayat C, Mahmoudi R, Chong EC, Tang W, Puentes G, Kang X, Abrahamson D. Does coronary artery screening by electron beam computed tomography motivate potentially beneficial lifestyle behaviors?. Am J Cardiol 1996; 78(11): 1220-1223.

**Electronic Supplementary Material 4: Summary table indicating the following for each included systematic review: the number of trials included; nature of risk information; number of studies targeting/reporting self-efficacy and response efficacy; behavior change techniques; and effective of interventions**

| **Author (date)** | **Number of trials included** | **Nature of risk information** | **Number of studies targeting/ reporting self-efficacy and response efficacy?** | **Behavior Change Techniques (BCTs) used in included studies** | **Effectiveness of interventions** |
| --- | --- | --- | --- | --- | --- |
| **Bize (2012)** | 15 TOTAL  15 smoking | 2 imaging/ visual  8 carbon monoxide  3 genetic testing  7 spirometry | 4 reported self efficacy  2 reported response efficacy  1 targeted self efficacy  0 targeted response efficacy | 12 provide information on consequences of behavior to individual  9 provide information on consequences of behavior in general  4 goal setting (behavior)  2 fear arousal  2 motivational interviewing  1 stress management/ emotional control training  1 relapse prevention/ coping planning | 2/15 studies showed significant effect on smoking |
| **DeViron (2012)** | 5 TOTAL  5 smoking | 1 carbon monoxide  5 genetic testing | 2 reported self efficacy  1 reported response efficacy  0 targeted self efficacy  0 targeted response efficacy | 5 provide information on consequences of behavior to individual  4 provide information on consequences of behavior in general  1 goal setting (behavior)  1 motivational interviewing | Effect (2 to 6 month) on smoking RR 1.55 (95%CI 1.09 to 2.21)  Effect (last follow-up) on smoking RR 1.03 (95%CI 0.64 to 1.65) |
| **Hackam (2012)** | 4 TOTAL  4 smoking  1 diet  1 PA | 4 imaging/ visual | 0 reported self efficacy  0 reported response efficacy  0 targeted self efficacy  0 targeted response efficacy | 3 provide information on consequences of behavior to individual  1 provide information on consequences of behavior in general  3 fear arousal | Effect on smoking OR 2.24 (95%CI 0.97 to 5.19)  Effect on diet OR 0.78 (95%CI 0.22 to 2.85) |
| **Hollands (2010)** | 4 TOTAL  3 smoking  1 diet  1 PA | 3 imaging/ visual  1 carbon monoxide | 1 reported self efficacy  1 reported response efficacy  1 targeted self efficacy  0 targeted response efficacy | 3 provide information on consequences of behavior to individual  2 provide information on consequences of behavior in general  3 fear arousal | Effect on smoking OR 2.81 (95%CI 1.23 to 6.41)  Effect on fibre intake OR 0.36 (95%CI 0.08 to 1.53)  Effect on fat intake OR 1.84 (95%CI 0.51 to 6.71)  Effect on physical activity SMD 0.10 (95%CI -0.04 to 0.24) |
| **Marteau (2010)** | 7 TOTAL  5 smoking  2 diet  2 PA | 1 carbon monoxide  7 genetic testing | 3 reported self efficacy  2 reported response efficacy  1 targeted self efficacy  1 targeted response efficacy | 7 provide information on consequences of behavior to individual  4 provide information on consequences of behavior in general  1 goal setting (behavior)  1 motivational interviewing | Effect (<6 months) on smoking OR 1.35 (95%CI 0.76 to 2.39)  Effect (>6 months) on smoking OR 1.07 (95%CI 0.64 to 1.78)  Effect on diet OR 2.24 (95%CI 1.1 to 4.27)  Effect on physical activity OR (95%CI 0.59 to 1.80) |
| **Rodondi (2011)** | 9 TOTAL  7 smoking  6 diet  5 PA | 9 imaging/ visual | 0 reported self efficacy  0 reported response efficacy  0 targeted self efficacy  0 targeted response efficacy | 6 provide information on consequences of behavior to individual  2 provide information on consequences of behavior in general  1 goal setting (behavior)  6 fear arousal | 1/7 studies showed significant effect on smoking  4/6 studies showed significant effect on diet  1/5 studies showed significant effect on physical activity |
| **Smerecnik (2012)** | 5 TOTAL  5 smoking | 1 carbon monoxide  5 genetic testing | 2 reported self efficacy  1 reported response efficacy  0 targeted self efficacy  0 targeted response efficacy | 5 provide information on consequences of behavior to individual  4 provide information on consequences of behavior in general  1 goal setting (behavior)  1 motivational interviewing | Effect (overall) on smoking OR 1.16 (95%CI 0.77 to 1.76)  Effect (<6 months) on smoking OR 1.87 (95%CI 1.20 to 2.92)  Effect (>6 months) on smoking OR 0.68 (95%CI 0.57 to 1.30) |
| **Usher-Smith (2015)** | 3 TOTAL  3 smoking  2 diet  1 PA  2 alcohol | 3 numerical | 0 reported self efficacy  0 reported response efficacy  0 targeted self efficacy  0 targeted response efficacy | 3 provide information on consequences of behavior to individual  2 provide information on consequences of behavior in general  1 goal setting (behavior)  1 barrier identification/ problem solving  1 goal setting (outcome) | 0/3 studies showed significant effect on smoking  0/2 studies showed significant effect on diet  0/1 studies showed significant effect on physical activity  0/2 studies showed significant effect on alcohol |
